# Rationale and design of a randomized controlled trial examining the effect of classroom-based physical activity on math achievement

**DOI:** 10.1186/s12889-016-2971-7

**Published:** 2016-04-11

**Authors:** Mona Have, Jacob Have Nielsen, Anne Kær Gejl, Martin Thomsen Ernst, Kjeld Fredens, Jan Toftegaard Støckel, Niels Wedderkopp, Sidsel Louise Domazet, Claire Gudex, Anders Grøntved, Peter Lund Kristensen

**Affiliations:** Centre of Research in Childhood Health, Department of Sports Science and Clinical Biomechanics, University of Southern Denmark, Campusvej 55, 5230 Odense M, Denmark; Department of Learning and Philosophy, Aalborg University, Fredrik Bajers Vej 5, P.O. Box 159, 9100 Aalborg, Denmark; Department of Clinical Research, University of Southern Denmark, J.B. Winsløws Vej 19, 2., 5000 Odense C, Denmark

**Keywords:** RCT, Classroom-based PA, School children, Academic achievement, Executive Functions, Embodied cognition, Methods

## Abstract

**Background:**

Integration of physical activity (PA) into the classroom may be an effective way of promoting the learning and academic achievement of children at elementary school. This paper describes the research design and methodology of an intervention study examining the effect of classroom-based PA on mathematical achievement, creativity, executive function, body mass index and aerobic fitness.

**Methods:**

The study was designed as a school-based cluster-randomized controlled trial targeting schoolchildren in 1st grade, and was carried out between August 2012 and June 2013. Eligible schools in two municipalities in the Region of Southern Denmark were invited to participate in the study. After stratification by municipality, twelve schools were randomized to either an intervention group or a control group, comprising a total of 505 children with mean age 7.2 ± 0.3 years. The intervention was a 9-month classroom-based PA program that involved integration of PA into the math lessons delivered by the schools’ math teachers. The primary study outcome was change in math achievement, measured by a 45-minute standardized math test. Secondary outcomes were change in executive function (using a modified Eriksen flanker task and the Behavior Rating Inventory of Executive Function (BRIEF) questionnaire filled out by the parents), creativity (using the Torrance Tests of Creative Thinking, TTCT), aerobic fitness (by the Andersen intermittent shuttle-run test) and body mass index. PA during math lessons and total PA (including time spent outside school) were assessed using accelerometry. Math teachers used Short Message Service (SMS)-tracking to report on compliance with the PA intervention and on their motivation for implementing PA in math lessons. Parents used SMS-tracking to register their children’s PA behavior in leisure time.

**Discussion:**

The results of this randomized controlled trial are expected to provide schools and policy-makers with significant new insights into the potential of classroom-based PA to improve cognition and academic achievement in children.

**Trial registration:**

Clinicaltrials.gov: NCT02488460 (06/29/2015)

## Background

Physical activity (PA) in schools has historically received little attention and, in many countries, economic concerns and a greater focus on standardized testing have reduced the time spent on physical activity in favor of academic classes [1–4]. A regular school day often includes very little PA and children are required to sit quietly in the same position for long periods of time [5–7]. However, this may not be the most effective way of enhancing children’s academic achievement. Breaking up the sitting periods with physical activity might be a better approach to promote learning [8, 9].

PA has several cognitive and physiological benefits [10, 11]. A meta-analysis showed a significant positive relationship between PA in general and cognition in children [12]. This is consistent with the “executive function hypothesis”, in which PA is believed to enhance executive functions that are crucial to the learning process, such as organization and integration of information, and modulation of behavior through selective attention (e.g. [13–16]). Studies report clear acute cognitive benefits of PA, typically increased information processing speed or response accuracy [17–19]. Early experiments on the effects of regular PA showed either no effect of PA on cognitive outcomes [20] or limited improvements [21, 22]. However, recent studies report positive effects of regular PA on the structures and functions of the brain [14, 23–25]. Possible neurophysiological mechanisms of PA include changes in brain blood flow and arousal level, improved conduction of information due to increased concentration of neurotransmitters and receptors, as well as increased concentrations of growth factors important for the development of new brain cells [26]. In addition, regular PA may increase brain volume (e.g. the volume of the anterior hippocampus), leading to improved spatial and relational memory [14, 27, 28]. Lastly, a recent study demonstrated a positive relation between mathematic achievement and decreased gray matter thickness in the superior frontal cortex, superior temporal areas, and lateral occipital cortex, in more fit children [29]. This suggests that aerobic fitness is essential for the normal thinning of the cortical gray matter during brain maturation, thus providing us with a predictor of math performance that could in turn help educators to identify interventions that can enhance learning.

Despite the increasing evidence for an association between PA or fitness and cognitive performance [30–35], few high-quality longitudinal studies have examined the effect of PA on academic performance (rather than cognitive control or other educational outcomes) [8, 14]. Furthermore, to the best of our knowledge, only three studies have assessed academic outcomes following the integration of PA into the classroom. A study conducted among US elementary school children reported that children with 90 min/week of moderate to vigorous physical activity in academic lessons over three years showed greater academic improvement than controls [36]. However, these results were considered secondary and were based on a small subsample (*n* = 203) of the study. Another small-scale randomized experiment from the US conducted among 3rd grade children showed that integrating 30 min of PA three days per week for three months gave mixed results for academic improvement compared with controls [37]. Finally, a recent study showed that physically active academic lessons significantly improved mathematics and spelling performance of elementary school children [38].

Even the studies investigating the link between PA and cognition have typically examined cognition and PA separately, possibly due to a commonly held view that the mind and body function separately. However, recent theories of embodied cognition have emerged in several disciplines to challenge this traditional dualistic approach [39–46]. These theories consider the body to be a key factor in shaping our cognition, where ‘non-cognitive’ processes of acting and perceiving providing bodily experience are fundamental to and inseparable from the development of important cognitive functions. This emphasizes the importance of sensory–motor interactions with our environment during learning, resulting in more endurable and richer consolidation of knowledge [47]. This psychological and neuroscientific research has implications for education as it emphasizes the importance of suitable sensory and motor interactions during learning for the development of human cognition [47]. 

We designed a randomized controlled trial with the primary aim of investigating the effect on mathematical achievement of incorporating physical activity in math teaching for 7-year-old schoolchildren. Secondary aims of the study were to investigate effects on executive function, and the longitudinal associations between PA and creativity, math, executive function, body mass index and aerobic fitness. The hypothesis was that PA integrated into classroom teaching would be superior to traditional, physically inactive classroom teaching in facilitating learning, and would be associated with a greater improvement in academic skills.

## Methods

### Study design

**Fig. 1:**
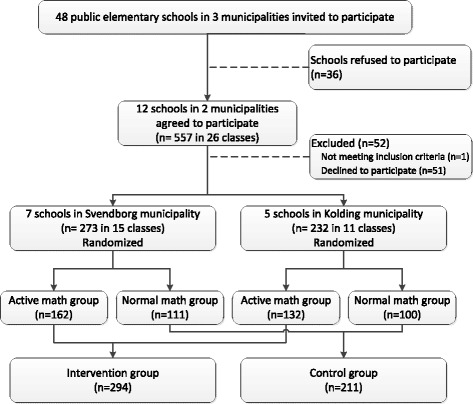
Flowchart of recruitment and randomization of schools in the study

The study was designed as a cluster-randomized controlled trial with two arms. In two municipalities in the southern part of Denmark, twelve elementary schools were randomized to either active math intervention (*n* = 7) or control (*n* = 5) (Fig. 1). Randomization was performed by random selection of sealed envelopes containing the intervention allocation stratified by municipality, in the presence of school leaders, municipality representatives and study researchers.

### Recruitment of participants

We approached three municipalities in the Region of Southern Denmark with the aim of recruiting schools and 1st grade children to the study. One municipality (15 schools) declined to participate. We hypothesized that children in this age group would particularly benefit from a classroom-based PA intervention, as they were likely to be struggling to sit in the same position in lessons for the majority of the day. Schools in these municipalities were eligible if they did not have a structured program that incorporated PA in the classroom.

Out of the 48 eligible schools that were invited to participate in the study, 12 schools accepted (7 in Svendborg municipality and 5 in Kolding municipality). The 1st grade children in these schools were invited to participate in a letter describing the study, measurement methods, randomization procedure and consent form. The children, parents and teachers were then invited to a meeting at the school, where a full description of the scientific background and the study was given. After this, 90.7 % of parents gave consent for their child to participate in the study, corresponding to 505 out of 557 students in 26 different classes. The excluded children received the same intervention as their classmates but did not participate in testing. A flowchart illustrating the recruitment and randomization process is shown in Fig. 1.

In Svendborg municipality, the participating schools were all part of an existing intervention study that had been initiated in 2008 (the CHAMPS-study DK). This intervention consisted of four extra lessons of physical education (PE) each week, in addition to the two compulsory lessons, resulting in a total of 4.5 h extra per week, divided over at least three weekly sessions of at least 60 min (a more detailed description of the CHAMPS-study DK can be found in [48]). To ensure that the extra PE lessons in Svendborg municipality did not bias the results of the present study, randomization to the intervention was stratified by municipality.

All schools were co-educational schools, with gender integrated classes in all subjects, which is typical for the Danish educational system.

Written parental consent was requested from parents in the included schools. Exclusion criteria were physical disability or no written parental consent. The trial protocol was approved by the ethics committee of the Region of Southern Denmark (S-20140105) and registered at clinicaltrials.gov (NCT02488460).

## The classroom-based PA intervention

The active math intervention consisted of math teaching that implemented PA in the classroom as a facilitating instrument. The intervention period lasted one school year, during which the students received on average 6 math lessons of 45 min per week with physically active teaching. PA in this math intervention was defined as any bodily movement produced by skeletal muscles that resulted in increased energy expenditure [49]. This is similar to the definition known as movement integration (MI) [50].

Each 45-minute lesson consisted of at least 15 min of PA spread over the lesson. Math lessons usually took place in the classroom, but were also held in other school areas such as corridors and surrounding outdoor areas. Sedentary activities were limited to periods of maximum 20 min, given that most students are unable to sustain attention on one thing for more than about 20 min at a time [51]. The rationale behind the active math was that if an abstract phenomenon is connected to a concrete bodily experience other than just seeing or hearing it, the comprehension and memory of the given phenomenon could be optimized (e.g. [52, 53]). In addition, we expected that breaking up the cognitive load imposed by a learning task would limit overload of working memory in the children and thus help maintain focus [54]. Studies have detected limited working memory capacity in children, and spreading the load across different working memory subsystems might prevent overload in one specific subsystem [55–57]. The active math lessons followed the principles of “learning by doing” and the development of practical life skills that are crucial to children’s education [58]. This is related to experiential learning theory, i.e. learning through action and experience as opposed to through repetition [59, 60]. The active math approach was adopted for all math lessons for the entire school year. Appendix 1 provides examples from the active math intervention.

Children in the control schools received regular classroom instruction, also with an average of 6 math lessons of 45 min per week. The math teachers in the control schools were asked not to make any changes to their usual teaching methods before the study endpoint measurements. This was accepted with the agreement that at the end of the study, these teachers would receive the same training in physically active math teaching as the teachers in the intervention group.

### Teacher training for the intervention

Teachers in the intervention schools attended a 4-day mandatory course on how to integrate active math into the Danish curriculum for mathematics in public schools. The aim of the course was to provide the teachers with the skills to implement physically active math lessons, i.e. to use suggested methods and develop new ways of integrating physical activity into lessons, to organize the activities and to report on intensity of PA (for an overview of the course plan, see Appendix 2).

The research group developed the training course and the associated training material that included specific instructions and inspiration for how teachers could integrate PA as an element of academic instruction in math lessons. Examples from this material were used to help illustrate the practical dimensions of the active math lessons. It should be noted that the focus of the course was on developing the teacher’s own abilities to design active math lessons and thus their own pedagogical skills and knowledge rather than handing out a complete manual with activities for the math lessons. The course included an introduction to the study context and aims, with emphasis on creating high motivation among the teachers for the intervention by explaining the numerous potential positive cognitive effects of learning through action and describing previous findings related to PA in general. The teacher’s motivation for implementing PA in the classroom and their involvement are highly relevant matters for math achievement [61], and could influence the outcome of the study. The teachers thus played an active role in creating and developing the activities for the math lessons. Furthermore, several theories of how to develop activities in the classroom were presented e.g. the ‘Activity-wheel’ [62], with the purpose of creating activities that would help the children to reach the desired learning objectives.

On the last course day, active teaching techniques were discussed and related to motivational, organizational and management techniques. Several teachers attending the course highlighted the challenges they expected to face when implementing PA in math. This initiated an internet-based intra-school communication forum in each of the two municipalities, where the teachers shared activities, experiences and advice for the active math intervention. The teachers received normal salary for attending the course, as compensation for their time.

## Baseline and follow-up assessments

The study was carried out over nine months from August 2012 to May/June 2013. Unfortunately during this period, all Danish schools were closed for a month due to a teacher lockout as a result of a union-employer dispute. Thus in the final period of the intervention, participants did not attend school for 25 days during April/May 2013. This period ended 4 weeks prior to the end of the intervention.

The test battery included measurements of math skills, cognitive function, creativity, aerobic fitness, anthropometrics and level of PA. Prior to testing, the research assistants (i.e. master’s and PhD students) were thoroughly trained in conduction of the test battery to ensure valid and uniform data collection. The training included several test trials with feedback and was performed at the University of Southern Denmark by a researcher experienced in the techniques. The research assistants were blinded to the randomization result for measurement of the outcomes and for data entry. All tests were performed at the participating schools either in the classrooms or a gym, except for the assessment of executive function (Flanker task), which was performed in smaller rooms with a maximum of two students present at the same time. Each outcome measure was assessed at baseline and follow-up using the methods described below. The tests applied at baseline and follow-up were identical.

### Mathematical skills test

Math skills were evaluated using a 45-minute standardized math test (MG) that was specifically designed for this age group by the developer of the Danish national tests (Hoegrefe Forlag) [63]. It consisted of 24 tasks assessing calculus and math in terms of the understanding of quantity and numbers, relations, addition, subtraction and geometry, with the final score ranging between 0 and 24. MG is a common test material in Danish primary schools and has been used for more than 25 years [63]. It is based on multiple-choice questions, and the different response options were selected in such a way that incorrect options related to typical mistakes made by students in 1st grade. The test provides a score reflecting the participant’s math skills and was conducted individually with paper and pencil in a classroom, with no aids permitted. Following a general presentation of the test, the participants were presented with each task separately and given 1 min to answer. The number of correct answers was used for further analysis.

### Flanker test

Executive functions were identified as working memory, inhibition, and cognitive flexibility, which were evaluated using a computer-based modified Erickson Flanker task [64]. Modified versions of the Flanker task have been used in previous studies indicating that the test is sensitive enough to detect changes in cognitive function associated with exercise [65]. The tests were conducted with the child seated at a 15” laptop with an external keyboard on which the corner keys with right and left arrows were clearly marked.

**Fig. 2:**
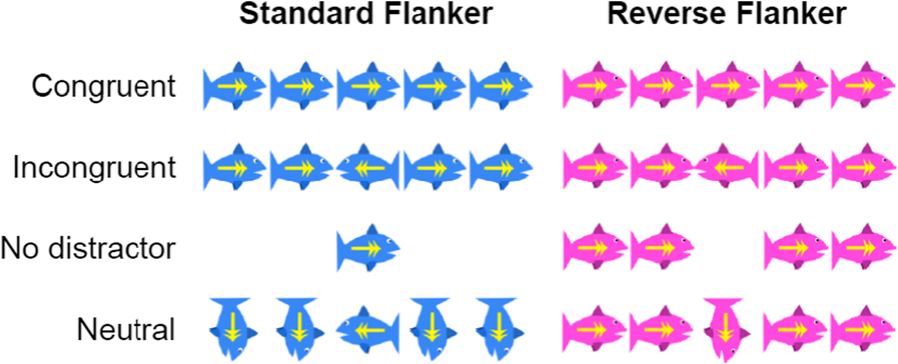
Stimuli in Flanker test for executive function. Examples of the four types of stimulus in the standard flanker (target stimulus: middle fish) and reverse flanker (target stimulus: ‘non-middle’ fish) conditions respectively

The task was to focus on a target stimulus ignoring other distracting stimuli. In this version, the stimuli were pictograms of fish, and the child was instructed to “feed” the fish by pressing the arrow in the direction the target stimulus (fish) was facing (Fig. 2). The test consisted of three conditions: (a) standard flanker, (b) reverse flanker, and (c) mixed trials. In the standard flanker condition, fish were blue and subjects were instructed to press the arrow that pointed in the direction the middle fish was facing (target stimulus), ignoring the distractor fish on either side. This task required remembering the rule for the task, paying attention to the task, and inhibiting distraction from the flanker fish on either side of the target stimulus. In the reverse flanker condition, the fish were pink. In contrast to the previous task, the child was now instructed to press the arrow that pointed in the direction the fish on either side (target stimulus) of the central fish were facing. Not only did this task require remembering the new rule for the task and selective attention, it also required cognitive flexibility to change from the strategy used for the standard flanker task. In the third condition of mixed trials, standard flanker (blue fish) and reverse flanker (pink fish) tasks were randomly intermingled, requiring flexible application of the rules for each task. This made a heavy demand on all three core executive functions and was expected to detect small individual and group differences.

The child was thoroughly instructed in the test by one of the research assistants, and made two familiarization rounds with blue and pink fish to ensure understanding of the task. Familiarization rounds consisted of four ‘habituation tasks’ where the child received verbal feedback from the test administrator, and then 17 further tasks. The child was instructed to respond as quickly and as accurately as possibly by pressing the left or right arrow on the keyboard. Each task was shown on the screen until an answer was given.

The test itself consisted of 45 tasks, with the first one not included in the analysis to exclude possible delay in the start-up. To test the child’s ability to focus and exclude interfering stimuli, the flankers were divided into four categories: 1) ‘congruent’ stimuli in which the flanking stimuli were identical to the target stimulus, 2) ‘incongruent’ stimuli in which the flanking stimuli were the opposite of the target stimulus, 3) ‘No distractors’ with no flanking stimuli and 4) ‘Neutrals’ in which the flanking stimuli were neither identical nor opposite of the primary stimuli but were vertically placed. The 44 tasks were divided equally between flanker conditions and contained 12 congruent, 16 incongruent, 8 no-distracter and 8 neutral tasks. These were also evenly divided between right and left as the correct answer. The stimulus presentation time was 1500 ms, the feedback interval was 1000 ms, and the interstimulus interval was 500 ms. The response time of the different tasks were recorded and the following variables were calculated: i) % accurate congruent answers, ii) % accurate incongruent answers, iii) the reaction time for correct congruent answers, and iv) the reaction time for correct incongruent answers. These parameters have previously been related to high levels of physical activity and fitness [66].

### BRIEF questionnaire

The Behavior Rating Inventory of Executive Function (BRIEF) is an assessment of behaviors at home for children and adolescents aged 5–18 years [67]. One parent or guardian for each child was asked to answer the questionnaire. After 3–4 weeks, parents were reminded to return the questionnaire via a message on the school’s intranet. Parents rated their child’s behavior on a three-point Likert scale (never, sometimes, and often). Eight scales were obtained (Initiate, Working Memory, Plan/Organize, Organization of Materials, Monitor, Inhibit, Shift, Emotional Control), along with a metacognition index (MCI), a behavior regulation index (BRI), and a global executive composite (GEC). The BRIEF Parent Form was normed on 1419 control children and 852 children from referred clinical groups; the Cronbach coefficient measure of internal consistency ranged from 0.80 to 0.98 for the parent and teacher form in clinical and normative samples [67].

### Creativity

Creativity was assessed because of a potential executive involvement in creative thought. We used the 30-minute standardized Figural Torrance Tests of Creative Thinking (TTCT): Thinking Creatively with Pictures, which requires the subject to reflect upon their life experiences. It is the most widely used test of creativity [68] and the most referenced creativity test [69]. The child was given three different activities and asked to draw and title one drawing to each task (pictures). Due to the mean child age of 7.2 ± 0.3 years, the Figural TTCT was chosen over the Verbal TTCT in liaison with the developer (Scholastic Testing Service) to avoid test results being influenced by writing skills. The Figural TTCT assesses the following mental characteristics: i) Fluency, a measure of the total number of interpretable, meaningful, and relevant ideas generated in response to the stimulus ii) Flexibility, related to the number of different categories of relevant responses iii) Originality, a measure of the statistical rarity of the responses, iv) Elaboration, related to the number of added ideas. The test was conducted according to the developer’s manual. It was completed individually with paper and pencil in a classroom, but was administered as a group test. After a general presentation of the test, the children were presented to each of the three tasks separately. Ten minutes were given to complete each activity.

In Activity I, the child constructed a picture using a pear or jellybean shape provided on the page as a stimulus. The stimulus was required to be an integral part of the picture construction. Activity II required the child to use 10 incomplete figures to make an object or picture. Activity III, was composed of three pages of lines or circles which the child should use as a part of the picture [70]. No aids were permitted.

### Aerobic fitness

Aerobic fitness was assessed using the Andersen test, an intermittent shuttle run test [71] that has previously been shown to be easy applicable and valid (*r* = 0.68) for measuring aerobic fitness in younger study populations. The children were instructed to run as far as possible in 10 min back and forth between two lines 20 m apart. This was performed in bouts of 15 s running followed by 15 s rest before the next bout. Participants were carefully instructed to take no more than two steps to stop after each bout of running and to sit down after the last bout facing the running direction to allow measurement of the distance of the last incomplete 2x20m round. Test personnel controlled the time and used a whistle to start and stop the participants. The test result was total distance in meters which has been validated against direct measures of maximal VO_2_-uptake [71, 72]. The Andersen test was found to be reproducible and valid (r2 = 0.53, *n* = 32, *p* < 0.001) for determining intermittent exercise capacity and estimating maximal oxygen uptake for children aged 6–9 years [72].

### Anthropometric measures

Body height was measured without shoes to the nearest 0.5 cm using a Harpenden stadiometer (West Sussex, UK). Body mass was measured in light clothing to the nearest 0.1 kg using an electronic scale (Tanita BWB-800, Tokyo, Japan). Body mass index was calculated as body mass (kg) divided by height (m) squared.

## Assessment of classroom physical activity and total physical activity

Intervention compliance was assessed using different methods targeting teachers and parents as well as the children. This included video analysis of lessons, SMS-tracking, and accelerometry.

### Video analysis

Math lessons were video-filmed for one week halfway during the intervention. The video camera (Mini DV DVR AVI Hidden Spy Video Camera & Voice recorder, AEE, China) was hidden to avoid behavioral change due to the camera attention. The teacher was asked to start and stop the camera at the beginning and end of the lesson.

### SMS-tracking

Short Message Service (SMS)-tracking was used to collect data from teachers about types and frequency of classroom PA elements. Each month, the math teachers received an SMS every day for one week asking about length, intensity of the PA (with specific examples of the three different intensities) and the teacher’s motivation for implementing PA in the math lessons. SMS-tracking was also used to assess the child’s leisure PA and school transport. This was accomplished by asking the parents to answer three questions. Parents received an SMS once a week during the entire intervention (see Appendix 3 for questions to teachers and parents).

### Classroom PA and total PA by students using accelerometry

The level of PA during math lessons and total daily PA were assessed using accelerometry (ActiGraph, GT3X and GT3X+, ActiGraph LLC, Pensacola, FL, USA). PA data were collected for eight days at baseline and again just before follow-up measurements. The accelerometers were randomly distributed by the research staff to all participants, who were instructed to wear the monitor during waking hours for at least eight consecutive days. Assessment periods were balanced between control and intervention schools. To eliminate reactivity bias, the monitors started recording the day after distribution. Epoch length was set to 2 s but subsequently downloaded in 10-s epochs. A sequence of more than 60 min of consecutive zeros was considered non-wear time and eliminated from the analysis. To account for children who failed to remove the monitor at night, only activity recorded between 6 am and 10 pm was analyzed. A valid measurement of total PA was defined as a minimum of four days with at least 10 h of recorded activity each day. For PA levels during math lessons only one valid day of measurement was required for inclusion in the analysis. Propero software (University of Southern Denmark, Odense, Denmark) was used to prepare the data for further analysis and to specify the time-points at which, according to the class schedule, math lessons took place. Total PA was expressed as mean counts per minute (cpm) and mean minutes in moderate-to-vigorous physical activity (MVPA) per day. Intensity cut-off points by Evenson et al. were used to define MVPA [73].

### Statistics

Descriptive statistics according to outcome measures and relevant baseline characteristics will be calculated and compared across allocation groups.

Using conventional two-sided levels of significance (0.05) and statistical power (0.8) and given a sample size of 550 children from 12 different schools, the minimum detectable difference in the change in math score between the intervention and control group will be 0.9 points, assuming a standard deviation of the change in math score of 3.6 points. If a school-level cluster effect of 0.07 is taken into account, the minimum detectable difference in the change in math score will be 1.9 points.

The effect of the intervention will be tested using mixed effects regression models adjusted for relevant potential confounding factors that were not randomly distributed in children from the intervention and control schools (i.e. gender, age, baseline value of the variable, socioeconomic status). Because of the clustered nature of the data, schools will be included as random effects in the analyses. All data will be entered twice and thereafter checked manually for outliers. Analyses will be performed with statistical software STATA version 14.

## Discussion

The literature lacks well-designed large-scaled randomized controlled trials on the effect of classroom-based physical activity on academic achievement and cognition. The present study will contribute to the field by generating detailed knowledge about the relationship between physical experiences that are combined with abstract math information and the acquisition of math skills. We expect the study results to provide schools and education policy-makers with an evidence base for implementing physical activity in daily school life, as a change from schoolchildren sitting still much of the day.

Apart from the large sample size and the comprehensive follow-up of children’s learning experiences and physical behavior over nine months, this study includes detailed measurement of the primary and secondary outcomes using well-validated methods. The math test applied in this study has been used for over 25 years to assess the math skills of Danish schoolchildren. The complex flanker task that assesses the three core executive functions will allow us to verify the few previous research findings concerning the association between a change in physical activity level and the ability to focus on a target stimulus and inhibit distractions [14, 74]. Furthermore, we expect the combination of SMS-tracking and accelerometry to be an excellent solution for measuring physical activity in large cohort studies. This makes it possible to compensate for some of the problems associated with accelerometry measurements, for example by using SMS-tracking to ask questions about specific activities, including water activities, and to allow more precise measurement of exercise intensity during cycling. When all this information is coupled to the class school timetable, then we can analyze the physical activity undertaken in specific time periods such as lunch breaks or during the math lessons. However, accelerometry does not provide any qualitative detail about the types or modes of activity being undertaken. For this reason accelerometry measurements were combined with video recordings of math lessons.

An important limitation of this study is the lack of qualitative data collection. This could have been used to further describe the various interventions and the complexity of the social contexts in which the active math was tested, (i.e. through case studies of a specific child/teacher or a specific context, observations of classroom activities, or qualitative interviews with children, teachers, and parents). Due to economic reasons, qualitative assessment was not performed since the main objective of the study was of quantitative character.

In conclusion, the results of this study will expand the current evidence on the relationship between physical activity and academic achievement in schoolchildren. We expect the results to stimulate the debate about whether the integration of physical activity into the classroom can enhance children’s cognitive skills and creativity, and will help educational practitioners to design learning environments that are optimal for cognitive development and academic achievement.

### Approval and registration

Ethics: Ethics committee of the Region of Southern Denmark (S-20140105)

Trial registration: clinicaltrials.gov (NCT02488460).

## Figures and Tables

**Fig. 3:**
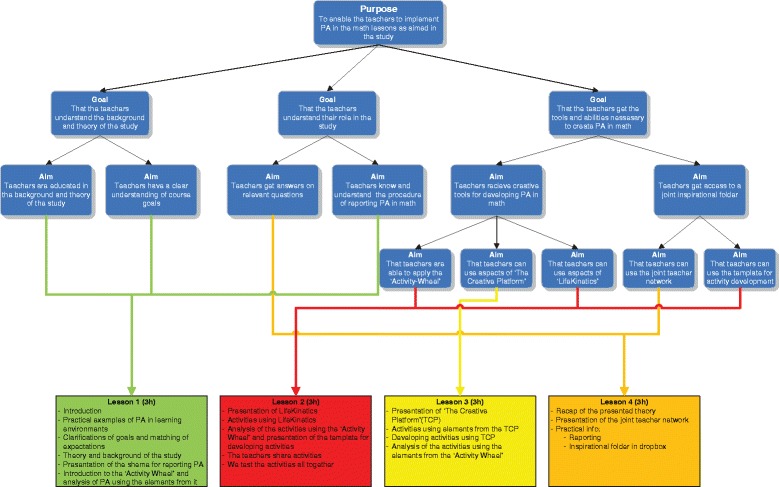
Teacher course goal hierarchy. Goal hierarchy explaining learning purpose, goals and aims for the teacher course on integrating movement into academic school lessons. The content is organized hierarchically so that moving down answers ‘how’ and moving up answers ‘why’. Thus goals answers how the purpose is achieved, and aims answers how goals are achieved. Furthermore goals answer why aims are relevant and should be achieved, and the purpose answers why goals should be achieved. Colored arrows depicts which aim that were in focus in the different workshops

## Appendix 1

### Active math intervention - Example 1: Mathematics darts

#### Aim

The aim of this activity is to rehearse simple mental arithmetic, i.e. to become familiar with numbers, addition, subtraction and mixed operations.

#### Materials

Balls or small sandbags to throw. Plates (approximately 1 x 1 m) with painted numbers and arithmetic operators (+, −, x,/), or you could draw these on the blackboard or outside on the floor/ground. The plates are divided into different sections (plus, times, minus, divide, etc. and various numbers 0 to 9).

#### Process

Students paired two and two are equipped with the balls and throw them one at a time at the numbers and operators. The aim is to be the couple who “reach” a predetermined number, such as “300” as fast as possible with the fewest throws. If the first of a pair of students throws three balls and hits “4”, “9” and “time sign”, they can form the equation 4x9 = 36, and have earned 36 points. If the student only hits numbers and no operators, then it can be agreed that the numbers must be added, or that no points are achieved in that round. The second student then takes their turn, and they continue to alternate until someone reaches the agreed total score.

#### Variation and progression

This task is quite versatile and the students can work with elementary multiplication and simple division or they can work with more complicated math questions, decimals and fractions. They can also bring paper or chalk to write down the calculations and remember them.

- The operator signs can be varied (e.g. plus, minus, multiply, divide, power, square root, parentheses)
- The numbers on the plates can be varied (e.g. natural numbers, integers, fractions, decimals)
- The students can do the task individually, in pairs, or in groups with or without timed competition
- The number of balls thrown in each round can be varied.

### Active math intervention - Example 2: Skipping rope

In this activity, the children are divided into pairs and they skip calculations to each other. One child skips while the other counts. If the child skips on two legs, one skip counts for 10, and a one-legged skip counts for 1. Thus if a child jumps three times with both legs and two times with one leg, the answer will be 32. This activity can also be made with ‘minus’, by skipping backwards and ‘multiply’ by skipping once with arms crossed.

## Appendix 3

### Short message service (SMS)-tracking for teachers

- How many times have you started an activity where the students have gone from sitting still to being active (that is, where they get away from their chairs)?
- How many minutes has the class been active at low, moderate and high intensity?

One Friday every month, the teachers answered the following questions on motivation:

- How important do you think physical activity is to promote students’ acquisition of math on a scale of 1 to 6, where 6 is most important?
- How motivated were you this week to implement physical activity in math lessons on a scale of 1 to 6, where 6 is extremely high motivation?

### Short message service (SMS)-tracking for parents

1. “How many times did [NAME OF CHILD] engage in sports during the last week”?

The parents were instructed to answer with the relevant number between 0 and 8. The answers 0 to 7 reflected the unique number of times engaging in sports, while 8 stood for ‘more than 7 times’.

2) Which type of sports did [NAME OF CHILD] undertake?

The parents were instructed to answer with a number between 0 and 10. The answers 0 to 9 each represented a unique type of sport, while 10 stood for ‘something else’.

3) How many times has [NAME OF CHILD] cycled to school in the last week?

The returned answers were automatically recorded and inserted into a database. To improve compliance rates, the responders were contacted by telephone if the answer did not fit with the instructions. Furthermore, a reminder was automatically sent if a participant had not responded within 72 h and, if necessary, again within 120 h of receiving the message.

## Abbreviations

BRI: behavior regulating index
BRIEF: behavior rating inventory of executive function
cpm: counts per minute
GEC: global executive composite
IMK: Ib Michael Kristiansen Almene Foundation
kg: kilograms
m: meters
MCI: metacognition index
MG: matematik grundlæggende
MI: movement integration
msec: milliseconds
MVPA: moderate-to-vigorous physical activity
PA: physical activity
PE: physical education
RT: reaction time
SD: standard deviation
SMS: short message service
TTCT: torrance tests of creative thinking
